# Brazilian dialysis survey 2019

**DOI:** 10.1590/2175-8239-JBN-2020-0161

**Published:** 2021-01-29

**Authors:** Precil Diego Miranda de Menezes Neves, Ricardo de Castro Cintra Sesso, Fernando Saldanha Thomé, Jocemir Ronaldo Lugon, Marcelo Mazza Nascimento

**Affiliations:** 1 Universidade de São Paulo, Faculdade de Medicina, Hospital das Clínicas, São Paulo, SP, Brasil.; 2 Hospital Alemão Oswaldo Cruz, São Paulo, SP, Brasil.; 3 Universidade Federal de São Paulo, São Paulo, SP, Brasil.; 4 Universidade Federal do Rio Grande do Sul, Porto Alegre, RS, Brasil.; 5 Universidade Federal Fluminense, Niterói, RJ, Brasil.; 6 Universidade Federal do Paraná, Curitiba, PR, Brasil.

**Keywords:** Censuses, Kidney Failure, Chronic, Epidemiology, Renal Dialysis, Peritoneal Dialysis, Brazil, Censos, Falência Renal Crônica, Epidemiologia, Diálise Renal, Diálise Peritoneal, Brasil

## Abstract

**Introduction:**

National data on chronic dialysis treatment are essential for the development of health policies that aim to improve the treatment of patients.

**Objective:**

To present data from the Brazilian Dialysis Survey 2019, promoted by the Brazilian Society of Nephrology.

**Methods:**

Data collection from dialysis units in the country through a completed online questionnaire for 2019.

**Results:**

314 (39%) centers responded the questionnaire. In July 2019, the estimated total number of patients on dialysis was 139,691. Estimates of the prevalence and incidence rates of patients undergoing dialysis treatment per million of the population (pmp) were 665 and 218, respectively, with mean annual increases of 25 pmp and 14 pmp for prevalence and incidence, respectively. The annual gross mortality rate was 18.2%. Of the prevalent patients, 93.2% were on hemodialysis and 6.8% on peritoneal dialysis; and 33,015 (23.6%) on the waiting list for transplantation. 55% of THE centers offered treatment with peritoneal dialysis. Venous catheters were used as access in 24.8% of THE patients on hemodialysis. 17% of the patients had K ≥ 6.0mEq/L; 2.5% required red blood cell transfusion in July 2019 and 10.8% of the patients had serum levels of 25-OH vitamin D < 20 ng/mL.

**Conclusion:**

The absolute number of patients, the incidence and prevalence rates in dialysis in the country continue to increase, as well as the percentage of patients using venous catheter as dialysis access. There was an increase in the number of patients on the list for transplantation and a tendency to reduce gross mortality.

## Resumo

**Introdução:**

Dados nacionais sobre o tratamento dialítico crônico são essenciais para a elaboração de políticas de saúde que almejem melhora no tratamento dos pacientes.

**Objetivo:**

Apresentar dados do Inquérito brasileiro de diálise de 2019, promovido pela Sociedade Brasileira de Nefrologia.

**Métodos:**

Coleta de dados das unidades de diálise do país através de questionário preenchido *on-line* referente a 2019.

**Resultados:**

314 (39%) centros responderam ao questionário. Em julho de 2019, o número total estimado de pacientes em diálise foi de 139.691. As estimativas das taxas de prevalência e incidência de pacientes em tratamento dialítico por milhão da população (pmp) foram 665 e 218, respectivamente, com médias de aumento anuais de 25 pmp e 14 pmp para prevalência e incidência, respectivamente. A taxa anual de mortalidade bruta foi de 18,2%. Dos pacientes prevalentes, 93,2% estavam em hemodiálise e 6,8% em diálise peritoneal, e 33.015 (23,6%) em fila de espera para transplante. 55% dos centros ofereciam tratamento com diálise peritoneal. Cateter venoso era usado como acesso em 24,8% dos pacientes em hemodiálise. 17% dos pacientes tinham K ≥ 6,0mEq/L, 2,5% necessitaram de transfusão de hemácias em julho/2019 e 10,8% dos pacientes apresentavam níveis séricos de 25-OH vitamina D < 20 ng/mL.

**Conclusão:**

O número absoluto de pacientes e as taxas de incidência e prevalência em diálise no país continuam aumentando, assim como o percentual de pacientes em uso de cateter venoso como acesso para diálise. Houve aumento do número de pacientes na lista para transplante e tendência para redução da mortalidade bruta.

## Introduction

The Brazilian Society of Nephrology (SBN) holds the Brazilian dialysis census annually. Such a survey is carried out online at a national level, with the goal of gathering information on clinical-epidemiological aspects, data on therapy and criteria of adequacy, among other factors inherent to patients and the chronic dialysis program.^1^^-^ 10 Epidemiological and technical data gathered through this census are highly relevant tools to create health policies, also enable projects and strategies to improve the care of dialysis patients. Despite the problems inherent to research based on voluntary provision of information, a significant portion of renal care centers in Brazil has contributed to this initiative.

This paper presents data from the 2019 Brazilian Dialysis Census, and compares it with data from 2016-2018, bringing new information regarding serum levels of potassium and vitamin D, the need for blood transfusion and details about centers and peritoneal dialysis financing.

## Methods

### Data collection

From August 2019 to January 2020, there was a national survey involving dialysis centers registered in the Brazilian Society of Nephrology, with the aim of collecting and analyzing data from patients on regular dialysis. To this end, a questionnaire with questions about sociodemographic, clinical-laboratory and therapeutic variables was made available on the SBN website from August of 2019 to January of 2020.

Participation in the census was voluntary, and all dialysis centers were invited, by letter and e-mail, to answer the questionnaire and send their data electronically to the SBN. After the initial invitation, reminders were sent monthly to those who had not filled in their data by the collection deadline - January 31, 2020. During the survey period, the Chairs of the SBN regional offices were tasked with contacting the directors of dialysis centers in their respective regions and encourage them to participate in the census. At the end of the data collection period, the SBN board, to emphasize the importance of participation, again contacted the dialysis centers.

### Data analysis

The data provided by the centers were grouped, and do not portray individual patient information. Since the sample from the centers that responded corresponded to 39% of all active centers, which is a substantial percentage for a voluntary survey, for national estimates of the total number of patients and the prevalence rate. The sample was expanded considering that the units that did not respond had the same average number of patients (n = 173.5) as the units that responded. As this assumption may be inaccurate, for the prevalence calculations we used a variation of ± 5% in the average obtained in the calculation of non-responding units (n = 164.8 to n = 182.2). Similarly, for the incidence rates, the average number of new patients per unit was applied for units that did not respond. All other calculations of sociodemographic and patient characteristics, use of medications and laboratory tests were performed considering exclusively the data obtained in the studied sample. Data relating to mortality rates and incident patients on dialysis were for events in July 2019, and their averages were estimated for the year.

For the prevalence and incidence calculations, we obtained this data from the Brazilian Institute of Geography and Statistics (IBGE), based on the Brazilian population of July 2018 and data relevant to the different regions of the country. According to this institute, the Brazilian population in July 2019 was 210.14 million inhabitants. To estimate the proportion of patients who did not reach the recommended targets^9^^-^ 12 for the dialysis dose (Kt/V or urea reduction rate), serum levels of albumin, phosphorus, parathyroid hormone (PTH) and hemoglobin, pooled data were used. Most of the data were shown in a descriptive manner and refer to 2019, some of which were compared with data from previous years.

### Calculations performed in estimates

Estimated total number (N) of patients on July 1: N of patients in the sample/proportion of participating centers. Estimated global prevalence: Estimated total N of patients on July 1/Brazilian population on July 1 of the corresponding year, expressed per million inhabitants (pmp). In regional and state estimates of N and ratios, the data considered was restricted to specific regions or states. Estimated total N of patients starting treatment in the corresponding years: (N informed of individuals starting treatment in July x 12)/proportion of active participating centers. Estimated global incidence: Estimated total N of patients starting treatment/Brazilian population on July 1 of the corresponding year, expressed pmp. To carry out the estimated prevalence and incidence calculations by state, we considered only those where at least 30% of the centers answered the questionnaire.

The prevalences related to demographics, clinical, laboratory and medication variables were expressed in relation to the totals derived from the answers related to each of the factors investigated among the 54,488 patients treated at the participating centers.

Estimated total number of deaths in the corresponding years: (N of deaths reported in July x 12)/proportion of active participating centers. Crude mortality rate: Estimated total N of deaths in 2019/Estimated N of dialysis patients on July 1 of the corresponding year.

## Results

**Figure 1:**
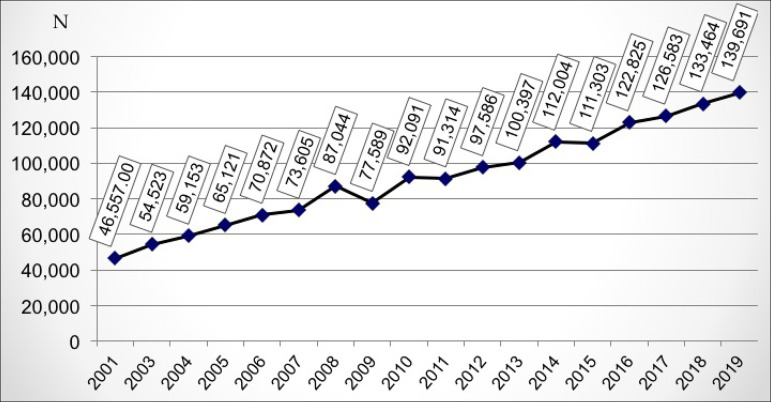
Estimated number of patients on chronic dialysis per year.

In July 2019, 805 centers maintained active chronic dialysis programs, an increase of 1.1% over the previous year, with this percentage increase also observed in relation to the number of centers that responded to the census (288 to 314). Such increase in adherence occurred mainly in the South and Midwest regions, which had, compared to 2018, an increase from 34% to 43% and from 27% to 34%, respectively. This increased compliance resulted in a 10.7% increase in the number of patients, whose information contributed to the data in the annual report (from 49,215 to 54,488), compared to 2018. In July 2019, the estimated total number of patients on dialysis in the country was 139,691. If the average number of patients estimated from the clinics that did not respond was 5% less or greater than that obtained in the sample of those who responded, the variation in the estimate would be from 135,240 to 143,766 patients. The trend towards a progressive increase in the number of prevalent patients in a chronic dialysis program was maintained (Figure 1), with an average increase of 6,881 patients (5.43%), compared to the last year.

Regarding the profile of dialysis clinics, there was a slight increase in the predominance of private clinics (71% to 73%), with a reduction in the percentage of non-university public services (86% to 84%), an increase in the percentage of satellite clinics (52% to 55%) and maintaining the public healthcare system - SUS - as the main paying source (79% of patients undergoing dialysis). There was a relative reduction of 2% in the percentage of clinics in the Southeast region (49% to 47%), with the same percentage increase found in the Midwest region (7 to 9%), and stability in other regions. There was also stability in the clinical occupancy rate (85%). The clinics participating in the census reported the presence of 2,072 active nephrologists; of which 1,967 (95%) had a medical residency or specialization validated by the SBN. However, there was no reduction in the average number of patients per nephrologist, which remained at 26, with an increase in the Northeast and Midwest (23 to 25 and 24 to 25, respectively) and a reduction in the North (33 to 31), the region with the highest patient/nephrologist ratio remains. There was a reduction of 4.7% (50% to 45.3%) in the proportion of nephrologists in the Southeast region in relation to the rest of the country, at the expense of a slight increase in all other regions. There was a reduction in the number of clinics that served patients with acute renal failure (75% to 69%), as well as that of patients undergoing conservative treatment of chronic kidney disease (84% to 78%). Regarding the time of machine use, there was an increase of 5% in the number of equipment with more than 6 years of use (44% to 49%), mainly to the detriment of those with 1-6 years of age (47% to 41%).

**Figure 2:**
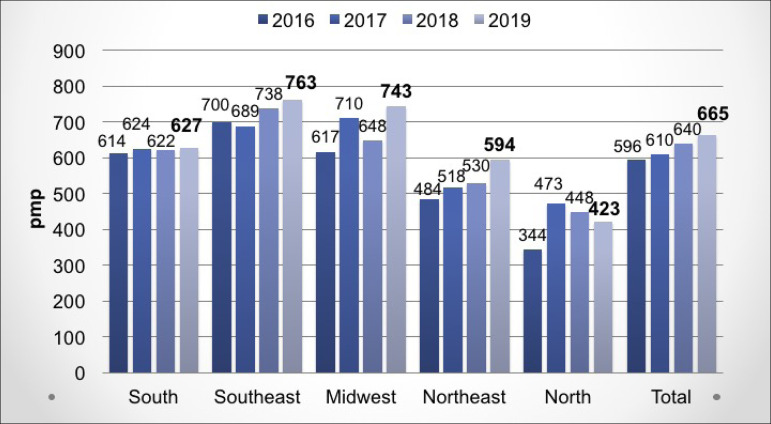
Estimated prevalence of patients on dialysis by geographic region in Brazil, 2009-2018 (per million of the population)

**Figure 3:**
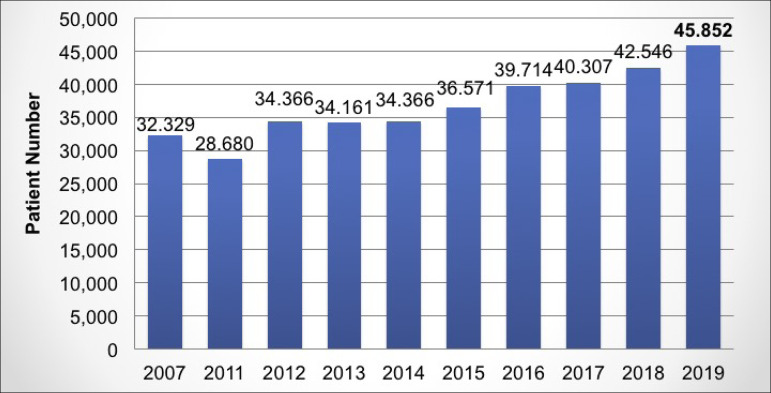
Estimated annual incidence of dialysis patients.

The estimated global prevalence of patients on chronic dialysis increased by 3.9%, from 640 to 665 pmp (range from 644 to 685 according to the number of patients estimated above), compared to 2018. Except for the Northern region (where there was a 5.6% reduction), in the other regions the prevalence rate increased, which was more evident in the Midwest and Northeast regions (14.6% and 12.1%, respectively), (Figure 2). The estimated number of new patients who started dialysis in 2019 was 45,852; an increase of 7.7% over the previous year (Figure 3), also seen in the estimated incidence rate, which was 218 pmp; 6.8% higher than in 2018. Table 1 depicts the estimated incidence and prevalence rates of dialysis patients in 2019. The states with the highest estimated prevalence rates of dialysis patients were Distrito Federal, Minas Gerais and Rio de Janeiro, with 942, 827 and 799 pmp, respectively; and the lowest rates were registered in Pará, Maranhão and Paraíba, with 384, 338 and 308 pmp, respectively. Regarding the estimated incidences, Goiás, Rio Grande do Sul and Paraná had the highest rates, with 291, 276 and 256 pmp; and the lowest incidences were recorded in Ceará, Maranhão and Paraíba (98, 77 and 60 pmp), respectively. The estimated annual incidence of new patients on dialysis for diabetic nephropathy was 79 pmp. Regarding the RRT method used, hemodialysis remains stable as the predominant method, 93.2% (an increase of about 1%). Peritoneal dialysis (PD), a method offered by 55% of the clinics that responded to the census, is a treatment modality used by 6.8% of the patients, about 1% less than in 2018. SUS (the Brazilian Public Healthcare System) is the main paying source, being responsible for financing 79% of PD patients. Among the available modalities, automated peritoneal dialysis (DPA) is the most frequently used (for 5.2% of the total patients), followed by continuous ambulatory peritoneal dialysis (CAPD) in 1.6%, with a reduction in the percentage of use of both therapies.

**Table 1:** Estimated incidence and prevalence rates of dialysis patients by state in 2019

UF	Incidence (pmp)	Prevalence (pmp)
AC	*	390
AL	*	*
AM	145	394
AP	*	*
BA	139	636
CE	98	566
DF	165	942
ES	192	680
GO	291	683
MA	77	338
MG	247	827
MS	*	*
MT	230	621
PA	116	384
PB	60	308
PE	191	645
PI	*	*
PR	256	692
RJ	246	799
RN	188	638
RO	*	*
RR	*	*
RS	276	639
SC	180	483
SE	*	596
SP	251	718
TO	*	422

* Note: estimate not done because of insufficient data.

**Figure 4:**
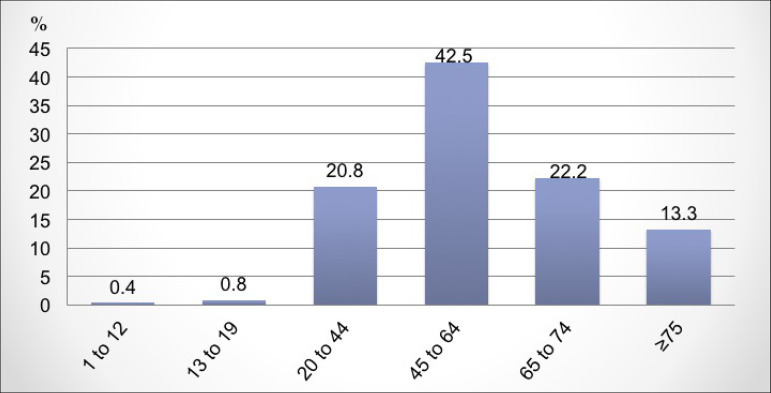
Distribution of patients according to age.

**Figure 5:**
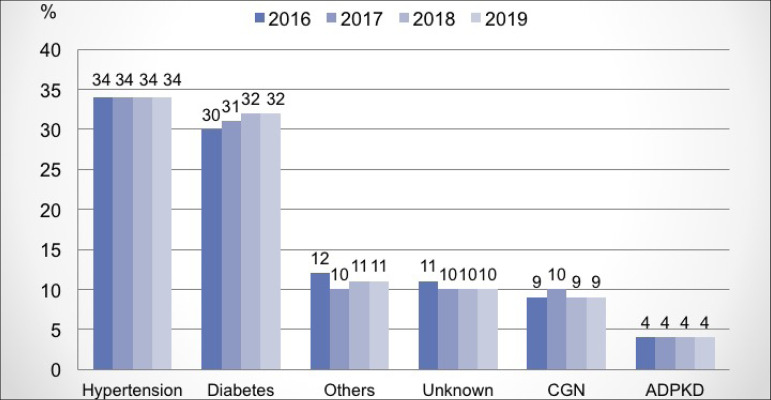
Distribution of dialysis patients according to underlying disease.

**Figure 6:**
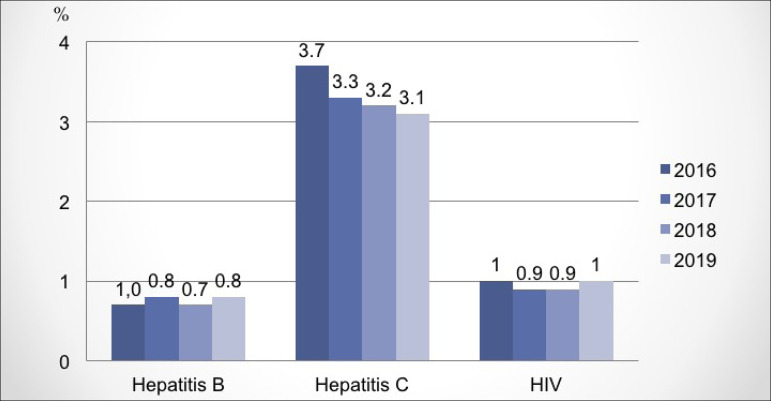
Prevalence of patients with positive serology for hepatitis B, C and HIV virus.

**Figure 7:**
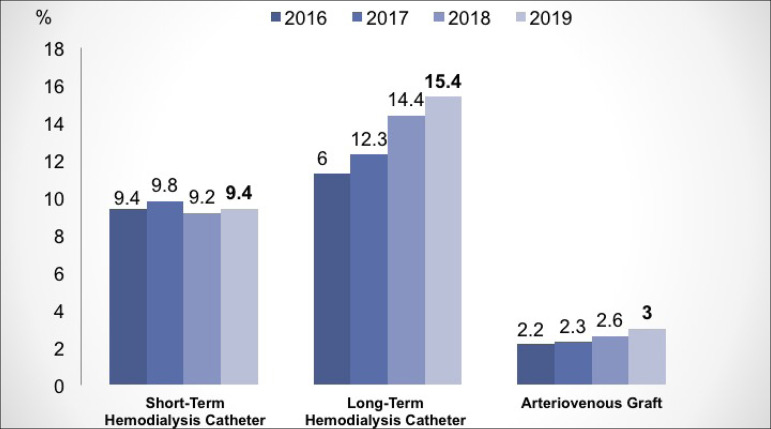
Distribution of vascular accesses used for hemodialysis.

**Figure 8:**
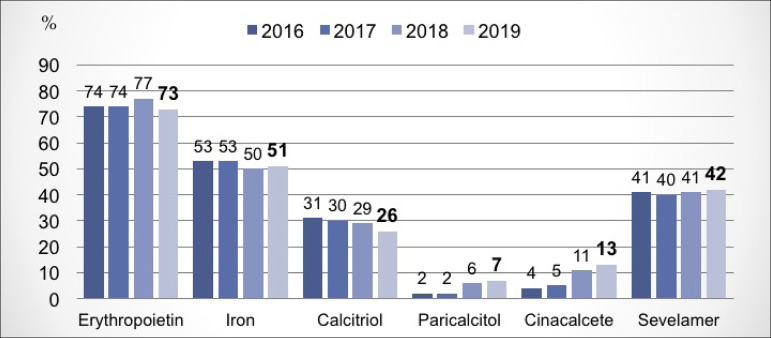
Pattern of medication use for the treatment of end-stage chronic kidney disease.

**Figure 9:**
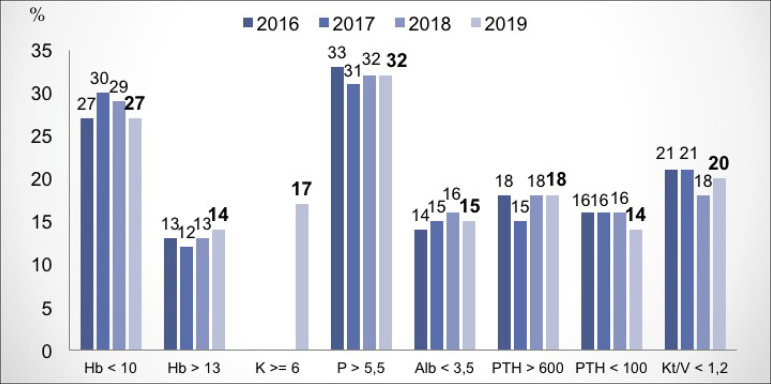
Proportion of patients with exams in non-compliance with indices recommended by the KDIGO.

**Figure 10:**
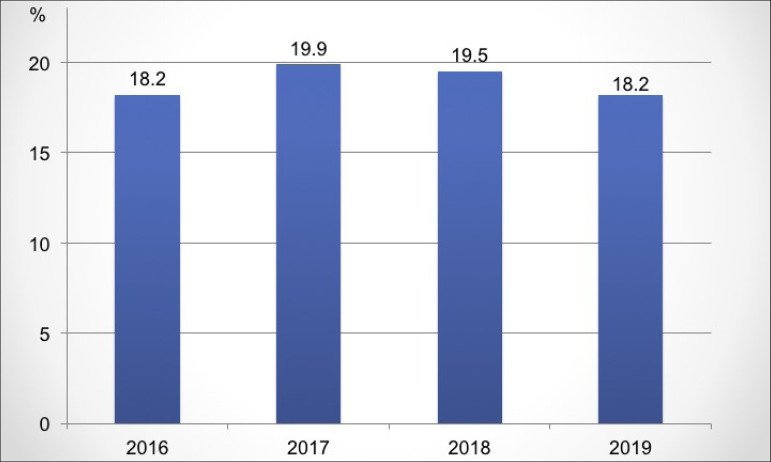
Estimated annual gross mortality rate of dialysis patients.

Regarding the profile of patients on dialysis, males represented 58%, and the age group between 45-64 years old, 42.5%; and 35.5% of the patients were over 65 years of age (Figure 4). With regards to the underlying disease, hypertensive nephrosclerosis remains the main cause (34%), followed by diabetic kidney disease (32%), with stable proportions in relation to 2018; with no variations in the proportions of the other causes (Figure 5). Regarding the body mass index (BMI), half (50%) of the patients have an adequate BMI (18.5-24.9 kg/m^2^), 8% below 18.5 kg/m,^2^ and 42% were overweight/obese (BMI ≥ 25 kg/m^2^), stable figures compared to last year. In the last three years, there has been a stability in the percentage of patients with positive viral serologies for hepatitis B, C and HIV, as shown in Figure 6. Regarding vascular access, there was a slight increase in the number of patients using long-term catheters (14.4% to 15.4%) and vascular prostheses (2.6 to 3%), with stability in the number of patients with short-term catheters (Figure 7). There was a 5% increase in the number of centers that reported using the same concentration of bicarbonate in the dialysis bath for all patients (78% to 83%), maintaining the median bicarbonate value in the bath at 32 mEq/L. Figure 8 shows stability in the figures in relation to the use of medications inherent to the treatment of renal failure. In the analysis of the dialysis adequacy parameters according to the KDIGO (Figure 9), there was an increase in the number of patients who did not reach Kt/V > 1.2, a decrease in the number with hemoglobin < 10g/dL and PTH < 100pg/mL. The other indexes remained stable. Since information is not available from previous censuses, 17% of the patients had K ≥ 6.0 mEq/L; 2.5% required red blood cell transfusion in July 2019; and 10.8% had serum levels of 25-OH Vitamin D < 20ng/mL. There was a percentage increase in patients using paricalcitol and cinacalcet (from 6% to 7%, and from 11% to 13%, respectively). There was no variation in the percentage of patients admitted per month, which remained at 5.8%. In 2019, the estimated number of patients on the waiting list for kidney transplantation increased by 11.7%, from 29,545 to 33,015, corresponding to 23.6% of dialysis patients; an increase of 1.5% in relation to the previous year. The estimated gross mortality rate was 18.2% (Figure 10). There was a drop in the estimated absolute number of deaths (from 25,986 to 25,481), which reflected a drop of 1.3% in the mortality rate, compared to last year.

## Discussion

Since 1999, the Brazilian Society of Nephrology has been collecting data for the Brazilian Dialysis Census.^1^^-^ 10 The purpose of this study was to outline the profile of patients on dialysis, and the reality of dialysis clinics with regards to the characteristics of the dialysis methods, issues inherent to the patient's clinical/epidemiological profile, adequacy in dialysis, vascular access, use of medications, mortality and transplantation; among others. The online format and the annual periodicity make it easier to fill in the data; however, compliance is still below the desired level: about 39% of the active centers. The estimated total number of dialysis patients in July 2019 was 139,691; of whom 79% were subsidized by the SUS, that is, 110,355 patients. This number is slightly higher than the number of cases billed by DATASUS in 2019. However, we must consider that DATASUS data also need validation and there may be an incorrect classification of patients.

In recent years, with increases in longevity, there has been a trend in Brazil^10^ to increase the number of prevalent patients on dialysis, a fact also reported from rest of the world.^11^^-^ 17 In the latest publication of the United States Renal Data System (USRDS),^15^ which brings data regarding chronic kidney disease and dialysis in the United States (USA) in 2017, there was a prevalence rate of 2,203 pmp. In its latest publication on dialysis and transplantation, the European Dialysis and Transplant Association (ERA-EDTA) Registry,^16^ which brings data from 2017, recorded a prevalence rate of 854 pmp. In relation to Latin America, 2018 data from the Latin American Society of Nephrology and Hypertension (SLAHN)^17^ show that the average prevalence rate of patients on renal replacement therapy (RRT), including dialysis and transplantation, was 805 pmp; and Brazil presented figures of 876 pmp. The highest rates were seen in Puerto Rico, Chile and Mexico (2,129, 1,541 to 1,405 pmp, respectively). The annual incidence rate of dialysis patients in Brazil in 2019 was 218 pmp, higher than last year, and also higher than the global rate in Latin America (154 pmp)^17^ and Europe (127 pmp)^16^. This rate, however, is lower than in other Latin American countries, such as Puerto Rico, Mexico and Honduras (419, 344 and 233 pmp, respectively), and in the United States (370 pmp)^15^. Hemodialysis is the most widely used dialysis method in Brazil (92.2%), as well as in Latin America (68.9%),^17^ In the United States it is 89.9%,^15^ and in Europe, 85%.^16^ A percentage of patients on peritoneal dialysis in Brazil has been progressively decreasing, following a trend also seen in Europe, the USA and Latin America, where this method is used for less than 10% patients.^10^^,^ 15 ^-^ 17

The analysis of the profile of patients prevalent on dialysis in Brazil reveals the predominance of men and a progressive increase in the age group of patients. As well as the increase in the prevalence of dialysis patients, the increase in the age group of dialysis patients can be explained by the greater burden of comorbidities, in addition to a substantial improvement in dialysis techniques.^10^ Such a change in the profile of patients on dialysis may require a review of care planning and dialysis treatment.^12^^,^ 14 ^,^ 18 ^,^ 19

Regarding the underlying diseases associated with CKD, arterial hypertension remains the main cause in Brazil,^10^ with diabetic nephropathy in second place. In the United States^15^ and Europe,^16^ as well as in the rest of the world, diabetes mellitus remains the main cause of CKD. Analyzing vascular accesses, there is still a proportion of patients with short- and long-term catheters, corresponding to about 25% of patients, higher than in the USA,^15^ where about 20% of patients use catheters. Regarding the assessment of hemodialysis adequacy parameters, there was a slight increase in the percentage of patients who did not reach the recommended values for Kt/V and who had Hb > 13g/dL. Nutritional parameters remained stable, but there was a decrease in the percentage of patients with Hb <10g/dL. Among the medications used to treat CKD complications, there was a reduction in the percentage of patients using erythropoietin and calcitriol, an increase in the use of paricalcitol and calcitriol, and stability in the use of the others.

The percentage of patients with positive serology for hepatitis B and HIV has remained stable in recent years, a trend also achieved by patients with hepatitis C, after a significant drop in prevalence in recent years, due to a reduction in transfusions, measures in relation to dialysis treatment itself (disposal of capillary and lines in all sessions), in addition to the availability of new treatments with a high rate of effectiveness.^20^^-^ 23

Compared to 2018, there was an increase of 11% in the percentage of dialysis patients enrolled in a transplant queue in Brazil, reaching the figure of 23.6% of patients; sharing with Uruguay the first position in Latin America.^17^ However, Mexico leads with the highest transplant rates, 79 pmp, higher than in Europe (33 pmp),^16^ and almost three times higher than Brazil, with a figure of 28 pmp. In the USA, 63.4% of dialysis patients were enrolled in a transplant list in 2017, and there is a reduction in the number of patients listed due to an increase in the absolute number of transplants in the country.^15^

In relation to 2018, there was a slight reduction (1.3%) in the gross mortality rate, which went from 19.5% to 18.2%. In recent years, the crude mortality rate in Brazilian patients on hemodialysis has remained between 15-20% per year, as well as the trend in other countries.^10^ In Europe, the 5-year survival of prevalent patients on dialysis (200- 2012) was 50.8%, which represents an average death rate of 9.84%/year, and in the USA, 165 per thousand patients.^15^

In the 2019 census, there was new information about peritoneal dialysis. Despite being a method not very much used in Brazil, it is offered by more than half of the dialysis clinics registered with SBN. In other words, the low use of PD does not seem to be related to the low availability of this method in the centers. In addition, our data show that 17% of the patients had serum potassium values above 6.0 mEq/L. Hyperkalemia in dialysis patients can reach figures of up to 37% of patients, increasing the potential risk of sudden death in the interdialytic period.^24^ In a meta-analysis that evaluated observational studies, having hyperkalemia increased the risk of cardiac mortality by 1.4 times.^25^ About 11% of the patients had 25-OH vitamin D <20 ng/mL, indicating that the vast majority of patients have an adequate serum level of this vitamin. Vitamin D deficiency is a frequent finding in patients with chronic kidney disease undergoing conservative treatment, and dialysis, reaching figures of up to 80% of patients.^26^ Replacement of such vitamins in dialysis patients was not associated with lower vascular calcification,^27^ and potential effects on mortality reduction are controversial.^26^

As limitations of the present study, we can mention the online data collection through voluntary filling, the grouping of patient data by dialysis center, the lack of validation of the responses sent and the insufficiency of information in some states. In addition, although the response rate of the centers was 39%, which is satisfactory for a voluntary survey, the methodology used in national estimates of prevalence and incidence rates is of limited accuracy, and should be interpreted with caution.

## Conclusion

The 2019 survey showed a continuous increase in incidence rates and prevalence of patients on dialysis. The inequalities between states and regions in relation to these estimates are evident, suggesting limitations in access to treatment. Despite the small percentage of patients treated for PD, 55% of clinics offer this type of treatment. The use of central venous catheters in hemodialysis patients continues to increase, as does the number of patients using paricalcitol and cinacalcet. About 17% of the patients had serum potassium ≥ 6.0 mEq/L, and 10.8% of the patients had serum 25-OH vitamin D levels < 20 ng/mL. Such data can assist in the establishment of strategies to improve the treatment of dialysis patients in Brazil.
